# Metabolomic mapping of cancer stem cells for reducing and exploiting tumor heterogeneity

**DOI:** 10.18632/oncotarget.21834

**Published:** 2017-10-15

**Authors:** Elisabet Cuyàs, Sara Verdura, Salvador Fernández-Arroyo, Joaquim Bosch-Barrera, Begoña Martin-Castillo, Jorge Joven, Javier A. Menendez

**Affiliations:** ^1^ Metabolism and Cancer Group, Program Against Cancer Therapeutic Resistance, Catalan Institute of Oncology, Girona, Spain; ^2^ Molecular Oncology Group, Girona Biomedical Research Institute (IDIBGI), Girona, Spain; ^3^ Unitat de Recerca Biomèdica, Hospital Universitari de Sant Joan, Institut d’Investigació Sanitària Pere Virgili, Universitat Rovira i Virgili, Reus, Catalonia, Spain; ^4^ Campus of International Excellence Southern Catalonia, Tarragona, Catalonia, Spain; ^5^ Medical Oncology, Catalan Institute of Oncology, Girona, Catalonia, Spain; ^6^ Unit of Clinical Research, Catalan Institute of Oncology, Girona, Spain

**Keywords:** cancer stem cells, metabolic maps, tumor heterogeneity

## Abstract

Personalized cancer medicine based on the analysis of tumors en masse is limited by tumor heterogeneity, which has become a major obstacle to effective cancer treatment. Cancer stem cells (CSC) are emerging as key drivers of inter- and intratumoral heterogeneity. CSC have unique metabolic dependencies that are required not only for specific bioenergetic/biosynthetic demands but also for sustaining their operational epigenetic traits, i.e. self-renewal, tumor-initiation, and plasticity. Given that the metabolome is the final downstream product of all the –omic layers and, therefore, most representative of the biological phenotype, we here propose that a novel approach to better understand the complexity of tumor heterogeneity is by mapping and cataloging small numbers of CSC metabolomic phenotypes. The narrower metabolomic diversity of CSC states could be employed to reduce multidimensional tumor heterogeneity into dynamic models of fewer actionable sub-phenotypes. The identification of the driver nodes that are used differentially by CSC states to metabolically regulate self-renewal and tumor initation and escape chemotherapy might open new preventive and therapeutic avenues. The mapping of CSC metabolomic states could become a pioneering strategy to reduce the dimensionality of tumor heterogeneity and improve our ability to examine changes in tumor cell populations for cancer detection, prognosis, prediction/monitoring of therapy response, and detection of therapy resistance and recurrent disease. The identification of driver metabolites and metabolic nodes accounting for a large amount of variance within the CSC metabolomic sub-phenotypes might offer new unforeseen opportunities for reducing and exploiting tumor heterogeneity via metabolic targeting of CSC.

## TUMOR HETEROGENEITY: CONCEPTS AND CHALLENGES

### Tumor heterogeneity: The greatest challenge in cancer therapeutics

The highly heterogeneous nature of cancer and the chaotic architecture of tumor tissues limit an accurate molecular classification, prognosis, and clinical response prediction for most human carcinomas. Intertumor heterogeneity encompasses the genetic and phenotypic variations that are observed between individuals with the same tumor type and between tumors of different tissues and cell types. Intratumor heterogeneity involves the variability that populations of tumor cells in a given tumor generally display in most discernable phenotypic traits, ranging from differentiation/proliferation states, migratory/invasive capacity, to therapeutic responsiveness. Tumor heterogeneity, which affects key cell- and non-cell-autonomous alterations that contribute to tumor evolution at any stage, may vary further over time if influenced by, for example, cancer treatment or metastatic dissemination. Tumor heterogeneity is ultimately responsible not only for the occurrence of multiple and distinct molecular subtypes associated with different clinical phenotypes and outcomes in a given cancer disease, but also for the ability of an individual tumor to survive therapy and seed metastases [1-10].

The extent of tumor heterogeneity confounds our understanding of tumor evolution and clinical progression and our ability to restrain treatment resistance and design effective cancer-therapeutic interventions. Tumor heterogeneity is beginning to be appreciated as the major obstacle to effective treatment and personalized medicine [11]. Although heterogeneity is assumed to arise from diverse cell types recruited to the tumor and from the differential integration of genetic, epigenetic, and microenvironmental influences among the cancer cells themselves [12-14], the ultimate mechanisms responsible for the emergence of tumor heterogeneity remain poorly understood and controversial. The current models explaining inter- and intra-tumoral diversity are the clonal evolution and cancer stem cell (CSC) hypotheses [15-18].

### Sources of tumor heterogeneity: Stochastic clonal evolution and genomic instability

The stochastic clonal evolution model posits that genetically (or epigenetically) distinct subclones arising through successive intercellular variations (e.g., chromosome copy number, somatic point mutations or epigenetic modifications) result in phenotypic diversity, followed by selective outgrowth of clones that have a phenotypic advantage within a given microenvironmental context. Although tumor evolution is apparently driven by selection of phenotypes according to their relative fitness, not all somatic mutations have a recognizable phenotypic consequence, and even fewer provide significant fitness advantages. Therefore, selection for phenotypic alterations can favor the outgrowth of cells with genetic alterations associated with that phenotype. At the intracellular level, a major cause of genetic heterogeneity is genomic instability [19, 20], which leads to an increased mutation rate and increased phenotypic variation, broadening the pool of cells that are subject to selection, and consequently the likelihood of selective expansion of multiple different subclones and the emergence of complex subclonal tumor architecture during disease progression. Fluctuations in subclonal architecture can occur in response to new microenvironments at metastatic sites together with the selection pressures imposed by the process of metastasis itself, or drug treatment.

### Cancer stem cells and cell plasticity: Key drivers of tumor heterogeneity

Tumor heterogeneity is generated through a combination of genetic alterations and epigenetic events that abnormally recapitulate normal developmental processes, including stem cell self-renewal and differentiation. Through comparisons with normal stem cell development, an ever-growing number of studies have established the existence of distinct subpopulations of so-called cancer stem cells (hereafter CSC), which are implicated as drivers of the origin, growth, and metastatic dissemination of most epithelial carcinomas. The aberrant capacity of CSC, also called tumor-initiating cells (TIC), for autorenewal and differentiation significantly contribute to the inter-tumor phenotypic and functional heterogeneity of a diverse array of cancer types and, by generating multiple, distinct cellular subpopulations in a tumor tissue, they have also emerged as key generators of intratumoral heterogeneity [13, 15, 17, 21-26].

The CSC model of tumor heterogeneity proposes that cancer cells residing in tumor tissues, although sharing similar genetic backgrounds, can be organized into two “operational” categories: CSC endowed with self-renewal and tumor-initiating potentials, and non-CSC. CSC therefore reside at the apex of the functional hierarchy within the tumor cell population as they possess the majority of a cancer’s tumor-initiating and metastatic ability. A defining feature of the CSC model is its apparent unidirectional nature, whereby CSC undergo symmetric division to replenish the CSC pool and irreversible asymmetric division to generate daughter cells (non-CSC) with low tumorigenic potential. However, evolving evidence supports a new model of tumorigenicity in which considerable plasticity exists between the non-CSC and CSC compartments, such that non-CSC can reacquire a CSC phenotype. The two broad functional classes of non-CSC and CSC do not necessarily reside in mutually exclusive subpopulations as cell plasticity allows phenotypic switching between non-CSC and CSC functional compartments. Indeed, CSC display significant phenotypic and functional heterogeneity and CSC progeny also manifest diverse plasticity, strongly suggesting that some tumors may adhere to a plastic CSC model in which bidirectional conversions are common and essential components of tumor heterogeneity [4, 16, 27-31].

### CSC-driven tumor heterogeneity: Mechanistic and therapeutic challenges

Since CSC can survive treatment with hormones, radiation, chemotherapeutic agents, and molecularly-targeted drugs, CSC-driven tumor heterogeneity might be responsible for the clinical failure of current oncology therapies [32-34]. If heterogeneity reflects hierarchical organization in which CSC irreversibly differentiate into non-tumorigenic cells, then therapies that eliminate CSC should be necessary and sufficient to cure disease. However, the appreciation of cancer cell plasticity as a mechanism that can generate aggressive CSCs within a tumor demand a radical revision of the earlier concept that only the self-renewal and tumor-initiating potentials of CSC need to be targeted to cure cancer.

If the heterogeneity within tumors reflects the reversible and efficient transition between CSC-tumorigenic and non-tumorigenic states, it might not be possible to identify any population intrinsically lacking tumorigenic potential. This new model implies that cancer therapies might not necessarily enrich cancer tissues with pre-existing, genetically-defined populations of treatment-refractory CSC, as previously thought; rather, accelerated de novo production of CSC from the residual cancer tissue may repopulate the tumor while the older CSC die. It would remain necessary to eliminate all cancer cells by combining anti-plasticity drugs with other cancer treatments since non-tumorigenic cells could drive disease recurrence by giving rise to new, heterogeneous populations of CSC [35].

### Understanding tumor heterogeneity: The next big challenge in cancer research

Understanding and exploiting tumor heterogeneity is the next big quest in cancer research [11]. Given that CSC may serve as the unit of selection in the genetic evolution of tumors while also being genetically unstable, the stochastic clonal evolution and CSC models are not mutually exclusive, and multiple clones consisting of genetically-altered CSC and their differentiated progeny can be generated during tumor evolution [9]. However, it should be noted that very different experimental and clinical predictions are expected to arise from cancer heterogeneity models in which intrinsic differences in tumorigenic capacity reflect reversible non-CSC/CSC as compared to those involving irreversible differentiation of CSC. In addition, the key contribution of the epigenome to tumor phenotype and clinical outcome is not generally incorporated into current models of CSC-driven tumor heterogeneity [36]. Furthermore, the epigenetic and genetic contributions to tumor heterogeneity are highly intertwined because genetic alterations can cause epigenetic disruptions while epigenetic defects can promote genomic instability.

It has been recently suggested that functional screening combined with multidimensional phenotyping–measuring signaling, epigenetic, transcriptional, and other alterations in addition to genetic alterations–will be most informative in revealing the sources of cancer heterogeneity and the contribution of heterogeneity to cancer evolution [11]. Unfortunately, many technological questions–including validation of the accuracy/robustness of gold-standard assays–regarding DNA, RNA, and protein measurements to obtain information about tumor heterogeneity remain unanswered. Moreover, we lack a clear understanding of the parameters that will need to be measured and integrated to assess the impact of tumor heterogeneity on clinical outcomes.

### Reducing the multidimensionality of tumor heterogeneity: The cell-state concept

One crucial question concerns the extent to which the phenotypic and functional properties of cancer cells including CSC, undergo reversible changes. To better approach the extreme complexity of the CSC model of cancer heterogeneity/plasticity, the concept of cell-state rather than genetically fixed cell-type would be applicable [11, 13, 37]. Cell states are defined by the interplay of the genome, epigenome, transcriptome and proteome in each tumor cell. Because cell states tend to be self-stabilizing, there are fewer distinct cell states in a tumor that the degree of genetic, epigenetic and transcriptional heterogeneity would predict. Genetically distinct cells may be in a similar cell state and hence may be susceptible to treatment with the same therapeutic. Conversely, genetically identical cells can exist in different cell states, owing to epigenetic differences and the influence of the microenvironment. An idoneous way to understand the enormous complexity of tumor heterogeneity would be to identify the most relevant cell states in cancer (such as those possessed by CSC) by integrating different data sets and, once these driver cell states are identified, work toward therapeutic strategies based on inferred cell states.

## CANCER STEM CELLS AND METABOLISM: A NOVEL APPROACH TO REDUCE AND EXPLOIT TUMOR HETEROGENEITY

To reduce the multidimensionality of tumor heterogeneity/plasticity into dynamic models of fewer actionable subtypes, we might require novel and innovative research approaches. We here propose that a novel approach to better understand the complexity of tumor heterogeneity is by mapping and cataloging small numbers of CSC metabolomic phenotypes.

### A CSC-metabolic framework: Experimental evidence

The last 5 years have witnessed significant advances in our understanding of how altered tumor metabolism, identified nearly a century ago by Otto Warburg [38, 39], is a central contributor to carcinogenesis rather than being a passive player [40-43]. At the same time, we have quickly amassed in-depth knowledge of the striking metabolic reprogramming phenomena that occur in pluripotent embryonic stem cells (ESC), tissue-specific adult stem cells (ASC), and induced pluripotent stem cells (iPSC) [44]. CSC also appear to exhibit unique metabolic features (BOX 1), which are required not only for supporting specific CSC bioenergetic/biosynthetic demands but also for epigenetically sustaining their operational properties, i.e. self-renewal, tumor-initiating, and plasticity potentials [44, 45].

### BOX 1. Metabolic traits of CSC states: From bioenergetic/biosynthetic features to metabolic regulation of epigenetics

#### CSC bioenergetic and biosynthetic features

The aberrant metabolic signatures of cancer tissues are not simply programmed consequences of oncogenic gain-of-function and loss of tumor suppressor mutagenic events; rather, they might play a pivotal role in dictating the different cell states exhibited by heterogeneous cancer cell populations. The modulation of metabolism has been increasingly implicated in cell identity determination during oncogenesis, i.e., metabolic reprogramming of cancer tissues might reflect the molecular dynamics fundamental to cell fate rearrangement. The occurrence of CSC states can be better understood in terms of the bioenergetic/biosynthetic facilitators and impediments that operate as molecular gateways and roadblocks, respectively, for the intrinsic and microenvironmental paths that ultimately orchestrate the CSC state [44-48].

A specific metabolic status involving changes in oxidative phosphorylation (OXPHOS)/glycolysis bioenergetics, mitochondrial-dependent biosynthesis, redox status, metabolism of amino acids and fatty acids, and in nutrient- and energy-sensing pathways, should become permissive with the operational properties owned by CSC; certain bioenergetic and biosynthetic features therefore become essential for maintaining CSC functionality. The cellular metabotype might causally govern key signaling determinants that ultimately determine the appearance, functioning, and potency of the operational properties possessed by CSC states. Conversely, the intrinsic and extrinsic genetic/epigenetic factors that control the path-to-CSC properties could not properly operate in inadequate cell metabotypes.

CSC states appear to exploit the siphoning of glycolysis- and/or mitochondrial-derived metabolic intermediates into de novo fatty acid biosynthesis to potentiate their self-renewal and survival capacities and escape detachment-induced cell death (anoikis) [49]. The dependence of CSC on the lipogenic activities of acetyl-CoA carboxylase (ACACA) [49] and fatty acid synthase (FASN) [50] might reflect a co-opted metabolic strategy to connect OXPHOS/glycolysis bioenergetic reprogramming with the intrinsic susceptibility of CSC to experience peroxidation phenomena and oxidative stress-induced cell death via regulation of the degree of saturated versus polyunsaturated acyl chains in CSC membranes. Accordingly, a promotion of pro-oxidant deviation using amine-pyrimidine-based iron complexes can efficiently kill epithelial-to-mesenchymal (EMT)-induced CSC-like states [51].

The EMT-driven switch from a non-CSC to a CSC-like state was found also to involve a nutritional metabolic infrastructure, allowing CSC states a vectorial energy transfer from a broader range of extracellular nutrients, including high-energy metabolites such as pyruvate and lactate, under stressful microenvironmental conditions [52]. The unexpected strong capacity of the anti-diabetic biguanide metformin to specifically target and eliminate CSC might largely depend on its ability to block the metabolic addiction of CSC states to the production of mitochondrial-dependent metabolic intermediates and the synthesis of nucleotides [53-58].

#### CSC metabolo-epigenetic features

Beyond the specific bioenergetic/biosynthetic demands of CSC, special classes of elite metabolites and the relative spatio-temporal abundance of common interpreters of the metabolic state that are critical factors for de/methylation, de/acetylation, or de/phosphorylation dynamics in the nuclear epigenome (e.g., acetyl-CoA, α-ketoglutarate, NAD+, FAD, ATP, or S-adenosylmethionine), might be causally involved in the redirection of normal and non-CSC toward a CSC-like state [46, 47]. We have coined the term metabostemness to describe the metabolic parameters that causally control the epi-transcriptional programs defining CSC states [35, 46-48, 59-62].

The appreciation that metabolites that act as cofactors for chromatin-modifying enzymes can directly influence the two primary epigenetic codes (histone modification and DNA methylation) to regulate many of the cell fate decisions has firmly established the notion that major metabolic pathways (e.g., one-carbon cycle, glycolysis, tricarboxylic acid cycle, and OXPHOS) can actively modify the chromatin state via largely unexplored metabolo-epigenetic axes of communication. Beyond the numerous “common” metabolites used as substrates and cofactors for reactions that coordinate epigenetic status, a recent systems approach predicted >40 compounds and metabolic substructures of potential oncometabolites (i.e., small-molecule components (or enantiomers) of normal metabolism whose accumulation is sufficient to establish a milieu that initiates and drives carcinogenesis [59-61]) that could result from the loss-of- and gain-of-function mutations of metabolic enzymes [63]. A metabolically driven corrupted version of the epigenome (e.g., pathological versions of nuclear reprogramming-like dedifferentiation phenomena [59-62]) might play an active role in directing CSC states in cancer tissues [62, 64].

A positive feedback loop might be established between the bioenergetic/biosynthetic demands and the epigenetically induced support of the CSC function and fate. Accordingly, the transcriptional activation of key nutrient- and energy-sensing pathways, i.e., mTOR and insulin receptor pathways, is an intrinsic process that occurs when differentiated populations of non-CSC breast cancer cells pass through dedifferentiating nuclear reprogramming-like processes to de novo development of CSC-like properties in vitro [65].

### CSC metabotypes: Built-in “barcodes” to detect and monitor CSC-driven cancer evolution

Certain metabotypes might operate as pivotal molecular events in the epigenetic and transcriptional rewiring required for the acquisition and/or maintenance of aberrant stemness and, concurrently, for the degree of refractoriness not only to different forms of cell death but also to differentiation. Because certain metabolic features appear to connect and integrate the functioning of all the –omic layers with self-autonomous but plastic CSC qualities, metabolic traits might have a particularly strong role in the definition of the CSC state and behavior at any given moment during cancer evolution and response to therapy. A highly active crosstalk between metabolism and epigenetics might allow the causal integration of certain metabotypes with genetic programs that coordinately regulate CSC function and fate. A progressive resetting of CSC-associated metabotypes would therefore parallel the bioenergetic/biosynthetic changes as well as the global epigenetic modifications of CSC states.

The mode of metabolic reprogramming in tumors (e.g., the best-known cancer metabolic abnormality termed the Warburg effect) is often considered a quasi-universal trait that differs from normal cell metabolism, displaying a wide diversity of metabolic phenotypes that have been suggested to reflect a function of both the genetic lesions driving tumorigenesis and the tissue from which the cancer arose [66]. Similar to epigenetic memory, we postulate that a metabolic memory might also exist –i.e., once a cell has passed through a particular metabolic state, some of the metabolic traits remain– thus influencing the functioning and plasticity potential of CSC originating from different tissues [46]. Thus, although the metabolic traits of CSC are expected to be dynamic, they might also represent the history of the cancer. The degree of flexibility in the metabotypic portraits of CSC might reflect the potential to respond to environmental or therapeutic pressures. The metabotypes of CSC states might be unique in their ability to provide information about the previous, present, and potential future of CSC-driven tumor heterogeneity and plasticity. Forthcoming studies would demonstrate that the metabolic features and dependencies of CSC states provide a built-in metabolic barcode that can be used to detect and monitor CSC functioning which, in turn, should significantly improve our ability to examine changes in CSC-driven tumor cell populations for cancer detection, prognosis, prediction/monitoring of therapy response, and detection of therapy resistance and of recurrent disease.

### The metabolome-CSC phenotype integration: Narrowing down the metabolomic diversity of CSC states to reduce multidimensional tumor heterogeneity

Given that metabolism represents a junction receiving cumulative information from multidimensional layers of signaling (e.g., genome, transcriptome, proteome, microenvironment), CSC states can adapt, resist, or react to all these multi-omic effects through a differential regulation, synthesis, and availability of certain metabolites. Indeed, all the –omic layers of complexity that drive CSC-driven tumor heterogeneity might concurrently generate CSC-associated metabotypes that can be described by means of at least four variability criteria: (a) the presence-absence of certain metabolites (e.g., oncometabolites); (b) the concentration levels of certain metabolites; (c) the relative levels or ratio between certain metabolites; and (d) metabolite profiles and fluxes. The strong occurrence of particular metabolic parameters in CSC states should be viewed as qualitative gauges necessarily and specifically stimulated by such particular CSC states or that lock CSC in such states by merely being present.

Although ostensibly simplistic, metabotypes based on the presence-absence of some particular metabolites can have high taxonomic value as sensitive and specific markers in distinguishing non-CSC from CSC states in cancer tissues. The increase in the concentration levels of some metabolites could also operate as dynamic markers governing the proclivity of non-CSC-to-CSC transitions. Metabolic ratios between the concentrations of structurally close metabolites can also provide functional discrimination between non-CSC and CSC states in heterogeneous cancer cell populations. Should CSC states possess specific variations in the capacities and kinetics of certain metabolic nodes, the discovery of unique, CSC-associated metabolic flux imprints might be crucial to delineate a comprehensive snapshot of the physiological state of CSC.

### CSC-metabolomic maps: Biological and therapeutic impact

Personalized health care of cancer patients requires a profound understanding of the patients´ biology that can be approached using a range of –omics technologies. The stratification of cancer patients should involve the identification of genetic and/or phenotypic disease subclasses that will require different therapeutic strategies. Stratified oncology medicine approaches to diagnosis, prognosis, and therapeutic response monitoring herald a new dimension in cancer patient care. Unfortunately, it has become exceedingly apparent that the utility of profiles based on the analysis of tumors en masse is limited by tumor genetic and epigenetic heterogeneity, as characteristics of the most abundant cell type might not necessarily predict the evolutionary properties of mixed populations.

**Figure 1:**
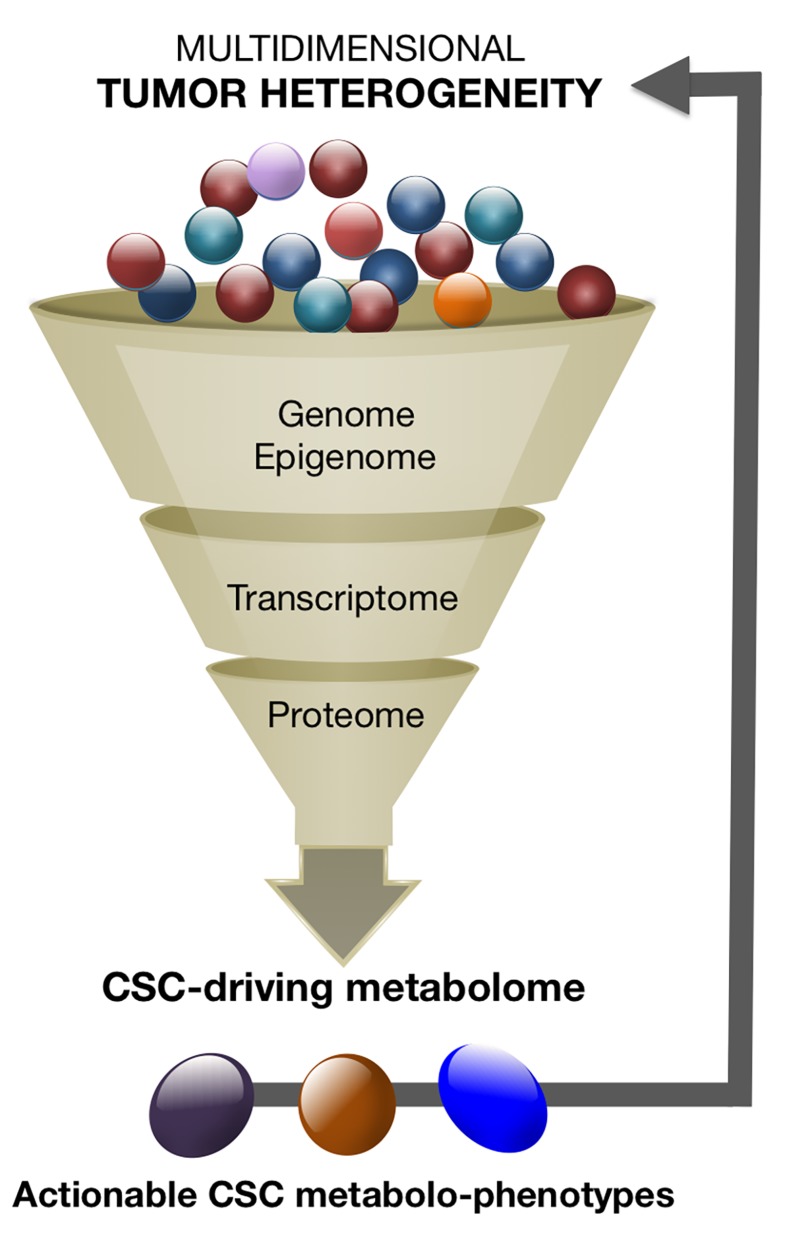
CSC metabolomics reducing and exploiting the high-dimensional complexity of tumor heterogeneity The narrow metabolomic diversity of the so-called CSC states could be employed to reduce multidimensional tumor heterogeneity into dynamic models of fewer actionable subtypes.

The metabolome is the final downstream product of all the –omic layers and, therefore, the most representative of the biological phenotype (Figure 1). Because metabolites are a proxy of the phenotype and the metabolome is a central hub for genetic and microenvironmental influences, i.e., the final result of the epigenetic reading of the genome in a particular environment that links the (epi)genotype with the phenotype, metabolomics-based approaches might have unprecedented value to bring clarity to complex –omics data. We envision that an unforeseen approach to better infer the enormous complexity of tumor heterogeneity is to catalog small numbers of more homogeneous CSC metabolomic phenotypes (Box 2).

### BOX 2. Deconstructing the CSC metabolo-phenome: Approaches and benefits

CSC states might exhibit unique metabolic features, which are required not only for specific CSC bioenergetic/biosynthetic demands, but also for epigenetically sustaining their operational properties, i.e. self-renewal, tumor-initiating, and plasticity potentials. As the metabolome is the final downstream product of all the –omic layers and, therefore, the closest to the biological phenotype, we propose that an unforeseen approach to better infer the enormous complexity of tumor heterogeneity is by cataloging small numbers of more homogeneous CSC metabolomic phenotypes, i.e., the sub-phenotyping of CSC metabolomic states:

1.) the narrow metabolomic diversity of CSC states can be utilized to reduce highly complex multiscale tumor heterogeneity into dynamic models of fewer, biologically and clinically relevant, actionable sub-phenotypes;

2.) the identification of key metabolic nodes sustaining self-renewal and chemoresistance of CSC states should provide new-targeted cancer-preventative and -therapeutic interventions; and,

3.) the exploration of the exo-metabolome to monitor biomarker/surrogate endpoints of CSC functioning and plasticity using liquid biopsies might optimize and accelerate therapeutic design of CSC-targeting personalized therapies.

Deconstructing the metabolo-phenome of CSC states might represent a new framework for reducing and exploiting the multidimensional complexity of tumor heterogeneity, also contemplates that integrating dynamic tracking of the same CSC metabolomic phenotypes in response to chemotherapy may allow for monitoring therapy responses as well as detecting early therapy resistance and recurrent disease at the level of CSC functioning. Moreover, CSC states are expected to generate CSC-specific metabonomic fingerprints that would be measured in the circulating exo-metabolome. The capture and analysis of the CSC-associated exo-metabolome as a new tool to monitor, in real-time, surrogate endpoints of CSC functioning and plasticity in liquid biopsies might optimize and accelerate therapeutic design of CSC-targeting personalized therapies.

We here propose to explore the potential value of metabolomic profiling as applied to CSC, a major unmet clinical need with no available specific treatments, to leverage the discovery of mechanistic information and deliver novel health care solutions to improve CSC-based clinical oncology. The sub-typing of CSC metabolomic states is expected to become an unforeseen strategy to reduce the dimensionality of tumor heterogeneity and improve our ability to examine changes in tumor cell populations for cancer detection, prognosis, prediction/monitoring of therapy response, and detection of therapy resistance and recurrent disease. Such proposal of CSC metabolic mapping might be explored using breast cancer as a paradigm of CSC-driven tumor heterogeneity (Box 3).

### BOX 3. Breast CSC: A paradigmatic model to explore CSC-metabolomic maps

Variations in CSC types across the spectrum of BC subtypes. Beyond the mutation profile as a source of genetic heterogeneity in the distinct molecular breast cancer subtypes, i.e., luminal A, luminal B, basal-liked, human epidermal growth factor receptor 2-enriched (HER2e), and claudin-low [67-71], a second level of epigenetic heterogeneity arises from the nature of the cells responsible for tumor maintenance and progression in each subtype, i.e., the so-called CSC or TIC. Despite the diversity of genetic changes driving the different molecular subtypes, two different types of CSC appear to exist in any of the breast cancer subtypes: a more proliferative, epithelial-like state characterized by the expression of the CSC marker aldehyde dehydrogenase (ALDH), and a more quiescent and invasive, mesenchymal-like state characterized by the expression of the CD44+CD24-/low immunophenotype [13, 72]. Remarkably however, each molecular breast cancer subtype is expected to display significantly different frequencies of epithelial CSC (E-CSC) and mesenchymal CSC (M-CSC). Thus, claudin-low and basal subtypes should contain a significant proportion of CD44+CD24-/low-expressing M-CSC, the HER2e subtype will be characterized by a high proportion of ALDH-positive E-CSC, the luminal B would contain a lower proportion of CSC than HER2e, basal, and claudin-low subtypes, and the luminal A subtype will display the lowest proportion of cells expressing any CSC marker [13, 69].

**Figure 2:**
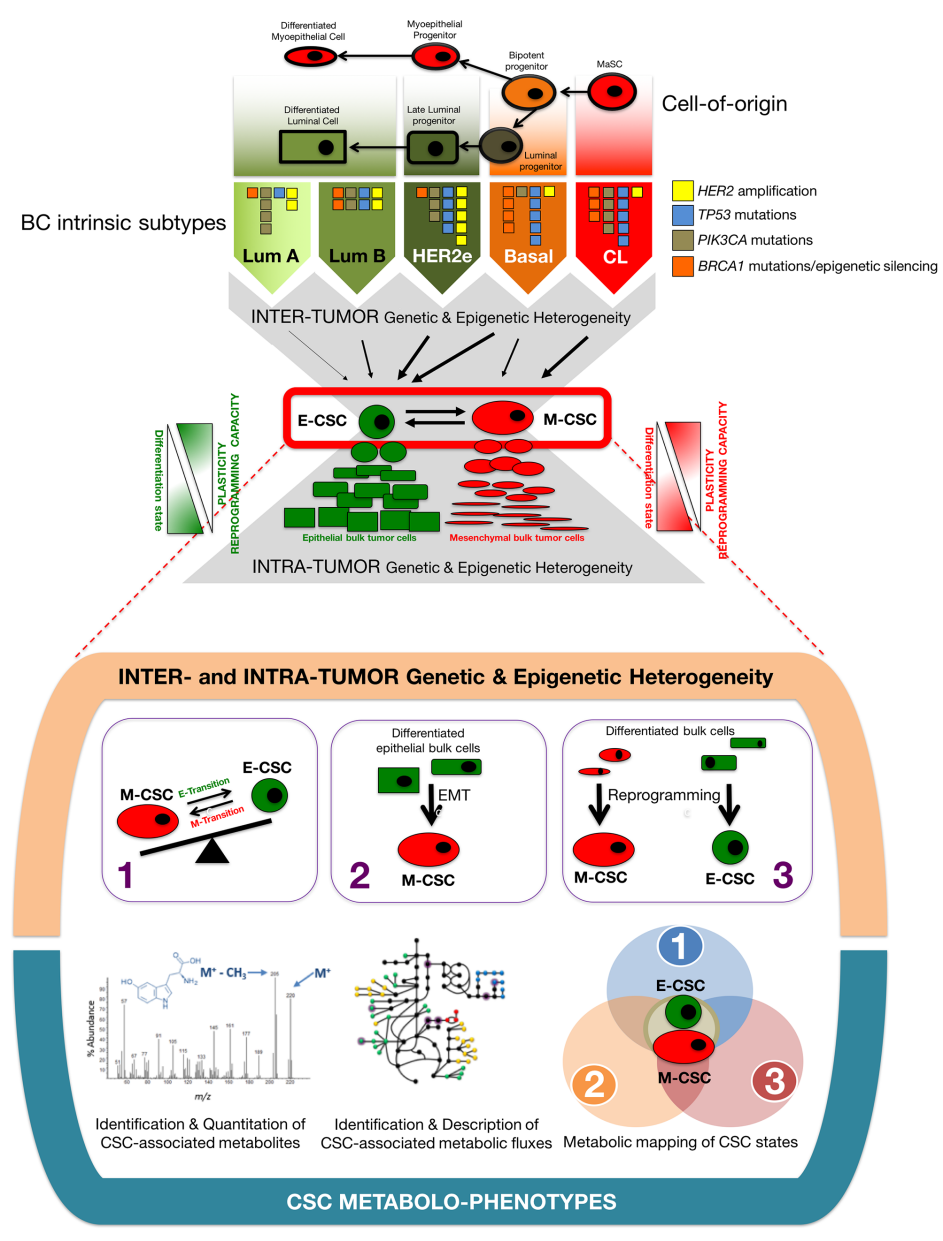
Breast cancer: A proof-of-concept test bed to connect CSC-driven tumor heterogeneity with discrete CSC metabolic maps Inter- and intra-tumor genetic and epigenetic heterogeneity in BC converge into a variety of intrinsically generated (1) and de novo (EMT- and reprogramming-) generated (2, 3) CSC states. CSC metabolo-phenotypes of discrete CSC states (e.g., ALDH+ E-CSC and CD44+CD24-/low M-CSC) are expected to represent central, operational metabolic hubs of tumor heterogeneity (modified from ref. [69]).

**Figure 3:**
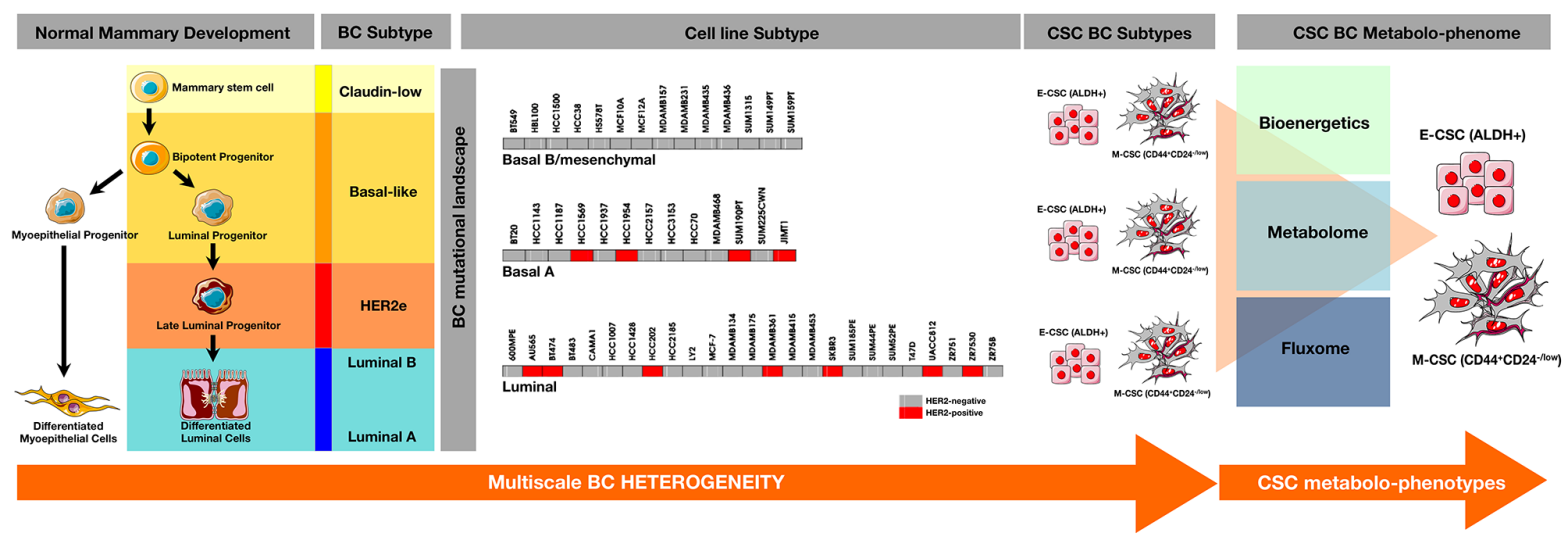
Comprehensive characterization of the CSC-associated metabolic traits across the spectrum of intrinsic breast cancer subtypes An enlarged catalog of the breast cancerpedia [73-79] including >50 breast cancer cell lines originally described by Neve et al. [73] as well as the so-called SUM-lines originally isolated by Prof. Steve Ethier [77-79] could provide sufficient heterogeneity and recurring characteristics at the genomic and transcriptional levels to recapitulate the characteristics present in intrinsic breast cancer subtypes. Such panel of breast cancer cell lines might be used to perform comprehensive studies of the bioenergetics, metabolome, and fluxomes of few groups of CSC metabolo-phenotypes capable of recapitulate the cellular heterogeneity of the original mixed population.

The fact that common, shared regulatory pathways are capable of directing self-renewal and differentiation of exclusively two major types of interchangeable CSC types irrespective of the different genomic/mutational landscapes of intrinsic breast cancer subtypes in which they reside, together with the ever-growing evidence that de novo generation of CSC-like states upon activation of EMT programs and nuclear reprogramming-like dedifferentiation phenomena might be activated in breast cancer as dynamic heterogeneity-generating mechanisms critical for the survival of tumors following therapy and metastatic progression, make breast cancer a paradigmatic model of tumor heterogeneity in which to perform a comprehensive metabolomic characterization of CSC states (Figures 2 and 3).

Breast cancer would allow to perform a comprehensive characterization of the CSC-associated metabolome in three different molecular scenarios known to generate a variety of CSC states: (1) The interchangeable populations of quiescent, mesenchymal-like CD44+CD24-/low and more proliferative, epithelial-like aldehyde dehydrogenase-expressing CSC, which convergently arise, although in different proportions, from the different genomic/mutational landscapes of intrinsic BC subtypes; (2) the EMT program that enables normal and non-CSC cancer epithelial cells to acquire CSC-like properties; and (3) the nuclear reprogramming-like phenomenon that might allow dedifferentiation of normal and differentiated bulk tumor cell types into CSC-like states (Figure 2).

**Figure 4:**
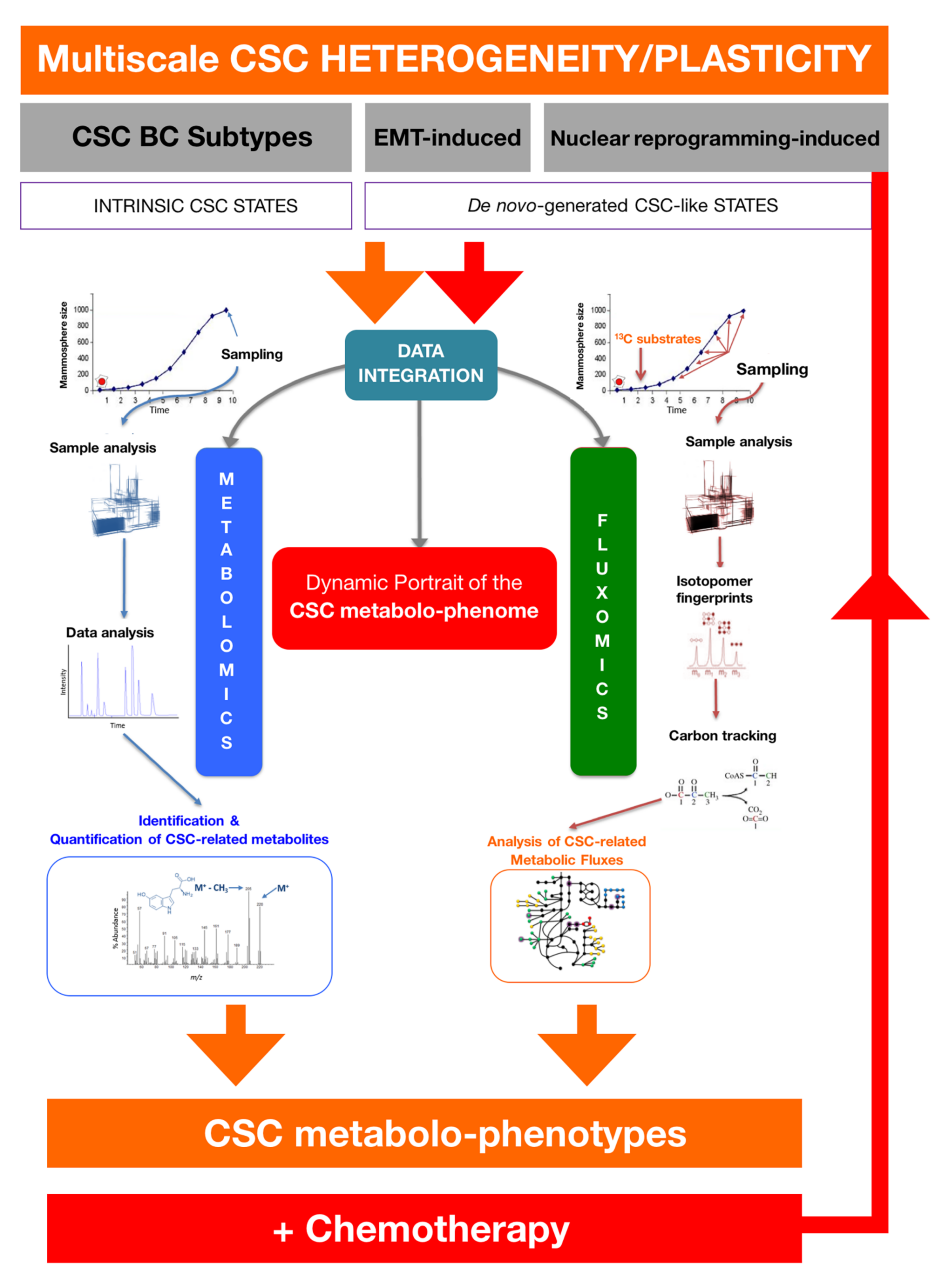
Deconstructing the CSC-associated metabolo-phenome The integration of metabolomics and fluxomics sciences by using exhaustive bioinformatics data analysis, mining and computation would provide a dynamic picture of the CSC-associated metabolic phenotype. The CSC-associated metabolic candidates and CSC-associated metabolic fluxes gathered in experimental approaches such as those depicted in Figs. 2 and 3. would be re-assessed in the presence of clinically-relevant concentrations of commonly used chemotherapeutic agents (e.g., anthracyclines, taxanes) employing representative scenarios of intrinsic and de novo-generated CSC states. The re-assessment of CSC-associated metabolomic fingerprints in response to chemotherapy would allow the identification of driver metabolic nodes accounting for a large amount of variance within CSC metabolomic phenotypes, which, in combination with the findings arising from the approaches depicted in Figs. 2 and 3 may provide not only a dynamic portrait of the CSC metabolo-phenome but also new CSC-targeted cancer-preventative and –therapeutic interventions.

The re-assessment of CSC-associated metabolomic fingerprints in response to chemotherapy would allow the identification of the metabolic networks and the driver metabolic nodes preferentially used by CSC states to regulate self-renewal and escape chemotherapy. The CSC-associated metabolic candidates and CSC-associated metabolic fluxes gathered would be re-assessed in the presence of clinically-relevant concentrations of chemotherapeutic employing representative scenarios of intrinsic and de novo-generated CSC states (Figure 4). This approach would allow to identify the driver metabolic nodes accounting for a large amount of variance within CSC metabolomic phenotypes, which, in combination with the metabolomic findings arising from treatment-naïve CSC states should provide not only a dynamic portrait of the CSC metabolo-phenome but also new CSC-targeted cancer-preventative and –therapeutic interventions. Moreover, the exploration of the microecology of CSC states in target tissues might generate specific and sensitive CSC-associated metabolomic fingerprints that could be measured in the circulating exo-metabolome. Such exploration of the exo-metabolome to monitor, in real-time, biomarker/surrogate endpoints of CSC functioning and plasticity in liquid biopsies might optimize and accelerate the design of CSC-targeting personalized therapies.

Our proposed approach might provide a first-in-class physiological snapshot of CSC states and actionable information to advance cancer research and clinical decision-making. The precise identification of the key metabolic parameters that directly communicate with the operational properties of CSC might uncover unexpected metabolic strategies to target life-threatening CSC in human cancer diseases. The incorporation of CSC metabolo-phenotypes as new parameters for reclassifying cancer subtypes and/or risk of therapy resistance and disease recurrence may provide a better and simplified dynamic delineation of the ever-growing number of cancer subtypes exclusively cataloged based on genetic aberrations. The discovery and development of new drugs targeting the metabolic nodes sustaining self-renewal growth of CSC states and CSC plasticity in response to chemotherapy could provide life saving treatments aimed to target those metabolic portraits compatible with the maintenance and/or acquisition of CSC operational properties, i.e., self-renewal, tumor-initiation, and plasticity.

The elaboration of CSC-metabolomic maps intrinsically accepts one of the cancer research field’s biggest challenges, namely the understanding of how aberrant versions of cellular metabolism and certain classes of metabolites operate as bona fide molecular hits enabling CSC states. We anticipate that such approach would illuminate crucial steps in a new era of metabolomics-based medicine to treat and eliminate CSC. Currently, the blood is viewed as the best source of information about the molecular makeup of tumor heterogeneity that can be obtained without biopsying the tumor itself. The development of non-invasive techniques such as circulating metabolomics capable of detecting and monitoring metabolites or metabolic imprints exclusively or differentially produced by CSC states could be rapidly implemented to provide real-time monitoring of CSC functioning by using blood-based liquid biopsies. Importantly, new approaches aimed to delineate CSC-metabolomic maps are clearly suited to becoming the platform for small or medium-sized enterprises dedicated to the exploitation of the underexplored field of CSC metabolism as they may allow cancer researchers to pursue first-in-class therapeutic and diagnostic approaches based on the addiction of CSC to certain metabolites or metabolic fluxes, thus opening a new era of metabolomics-based precision cancer medicine aimed to metabolically eliminate or prevent the occurrence of CSC states.
